# Genomic Studies of White-Rot Fungus *Cerrena unicolor* SP02 Provide Insights into Food Safety Value-Added Utilization of Non-Food Lignocellulosic Biomass

**DOI:** 10.3390/jof7100835

**Published:** 2021-10-05

**Authors:** Zichen Zhang, Aabid Manzoor Shah, Hassan Mohamed, Yao Zhang, Nino Tsiklauri, Yuanda Song

**Affiliations:** 1 Colin Ratledge Center of Microbial Lipids, School of Agriculture Engineering and Food Sciences, Shandong University of Technology, Zibo 255000, China; zichen_zc@163.com (Z.Z.); aabidshah@sdut.edu.cn (A.M.S.); hassanmohamed85@azhar.edu.eg (H.M.); zhangyao@sdut.edu.cn (Y.Z.); 2 Normal College, Jishou University, Xiangxi 416000, China; 3 Department of Botany and Microbiology, Faculty of Science, Al-Azhar University, Assiut 71524, Egypt; 4 Durmishidze Institute of Biochemistry and Biotechnology, Academy of Sciences of Georgia, Tbilisi 0159, Georgia; ninotsikla@mail.ru

**Keywords:** *Cerrena unicolor*, genome sequencing, lignocellulolytic enzymes, biomass degradation, carbohydrate-active enzymes (CAZymes)

## Abstract

*Cerrena unicolor* is an ecologically and biotechnologically important wood-degrading basidiomycete with high lignocellulose degrading ability. Biological and genetic investigations are limited in the Cerrena genus and, thus, hinder genetic modification and commercial use. The aim of the present study was to provide a global understanding through genomic and experimental research about lignocellulosic biomass utilization by *Cerrena unicolor*. In this study, we reported the genome sequence of *C. unicolor* SP02 by using the Illumina and PacBio 20 platforms to obtain trustworthy assembly and annotation. This is the combinational 2nd and 3rd genome sequencing and assembly of *C. unicolor* species. The generated genome was 42.79 Mb in size with an N50 contig size of 2.48 Mb, a G + C content of 47.43%, and encoding of 12,277 predicted genes. The genes encoding various lignocellulolytic enzymes including laccase, lignin peroxidase, manganese peroxidase, cytochromes P450, cellulase, xylanase, α-amylase, and pectinase involved in the degradation of lignin, cellulose, xylan, starch, pectin, and chitin that showed the *C. unicolor* SP02 potentially have a wide range of applications in lignocellulosic biomass conversion. Genome-scale metabolic analysis opened up a valuable resource for a better understanding of carbohydrate-active enzymes (CAZymes) and oxidoreductases that provide insights into the genetic basis and molecular mechanisms for lignocellulosic degradation. The *C. unicolor* SP02 model can be used for the development of efficient microbial cell factories in lignocellulosic industries. The understanding of the genetic material of *C. unicolor* SP02 coding for the lignocellulolytic enzymes will significantly benefit us in genetic manipulation, site-directed mutagenesis, and industrial biotechnology.

## 1. Introduction

*C. unicolor* has long been a traditional Chinese medicinal fungus and has been widely utilized to treat many human ailments in Asian countries. In the order Polyporales, it is a wood-decaying basidiomycete having ecological and biotechnological significance [1]. A large spectrum of enzymes that are directly or indirectly involved in the breakdown of organic wastes gives saprotrophic mushrooms their environmental and economic benefits [2]. White-rot fungi secrete a variety of intracellular and extracellular enzymes that degrade and utilize lignocellulose and naturally mineralize the lignin [3]. *C. unicolor* can produce high amounts of the enzyme without inductors [4], known as the potential bio-producer of industrially relevant enzymes such as laccase [5], manganese peroxidase [6], versatile peroxidase [7], cellobiose dehydrogenase [8], xylanase, and cellulase [9]. *C. unicolor* has a unique capacity to degrade recalcitrant resistant lignin polymers as well as a wide spectrum of aromatic pollutants that provide potential value in biomass utilization and organo-pollutant degradation [10,11]. The most abundant renewable biomass is lignocellulosic biomass, which has significant potential in the bio-refining industry [12]. Selective delignification by white-rot fungi like *C. unicolor* preferentially removes lignin from wood and leaves pockets of white degraded cells that consist entirely of cellulose [13,14], providing an eco-friendly way to obtain the high-value products and recycle the excess of agro-industrial waste [15]. In the context of *C. unicolor’s* abilities to degrade lignocellulosic biomass and production of biotechnologically significant compounds, better understanding of the inner relationship of lignocellulolytic enzymes and selective substrate utilize mechanisms may be extremely valuable.

*C. unicolor* is a virtual cellular “factory” that is widely known also as a valuable source of various biotechnologically applicable compounds [16]. *C. unicolor* as traditional medicine is now well-understood scientifically. Bioactive substances of pharmacological and medicinal relevance with antitumorigenic, antimicrobial, and antioxidative properties activity have recently been discovered [17,18,19]. Interestingly, laccases have also been reported with antiproliferative and pro-oxidant property [20,21]. Enzymes with bioenergy potential and secondary metabolites with therapeutic qualities have also been the subject of extensive research. [22]. 

The use of food-safety white-rot fungus such as *Ganoderma lucidum, Lentinus edodes,* and *Pleurotus ostreatus* for their nutritional and health-promoting characteristics has increased in recent years [23,24,25], and the unexploited wild basidiomycetes have gained significant attention in both academic and industrial areas [26,27]. However, there are still many limits on the commercialization and industrialization for basidiomycetes, even though they could have industrial, agricultural, medicinal, environmental, and socio-economic consequences [28]. Genome sequences are significant resources for genetic or molecular-based analysis, and a genome study of mushrooms has been done in several areas [29], which is advantageous for deciphering genetic diversity and genes affecting important traits [30,31]. The genome research of other non-model fungi is still in its infancy, although genomic studies of model organisms have received a lot of scientific attention [32]. Phylogenomic investigations of the genomes of wood degrading fungi have revealed information about the diversity and evolution of basidiomycetes’ lignocellulosic degradation [33], and diverse genomes are thus necessary for various research purposes. The genetic foundations and mechanisms of lignin degradation are major research focus areas in white-rot fungi study [34]. The growing number of genomes creates an opportunity to evaluate, compare, and formulate an optimal combination of biocatalysts with lignocellulolytic properties for primary and applied research. Genetic analysis provides new insights into *C. unicolor* genes related to diverse metabolic pathways and contributes to elucidating the utilization of the substrate as a source of energy for growth and colonization on lignocellulosic plant biomass and the metabolic changes induced in the fungus cell by variable environmental conditions [35]. 

Contrary to the numerous studies on bioactive substances, minimal attempts have been undertaken to investigate *C. unicolor* metabolic capacities and nutritional properties genetically. The genome-scale compositions of CAZymes and oxidoreductases operating on lignocellulose in this fungus are unknown. To date, only the *C. unicolor* 303 genome has been publically published under the Cerrena genus. The entire genome has been annotated by the JGI (http://genome.jgi.doe.gov/programs/fungi/index.jsf, accessed on 18 December 2017) and annotated with data from the ESTs. However, no additional study was published on *C. unicolor* 303. As a result, little was known about the genes involved in lignocellulose degradation and the molecular process used by Cerrena species to decompose wood. Due to a lack of genomic information, the development and exploitation of industrially valuable genes have been hampered, as well as the ability to manipulate them. Therefore, in the present study, the genome of *C. unicolor* SP02 was de novo sequenced and assembled with the combination of Illumina and PacBio sequencing strategies. Then, we did gene function analysis and annotated genes that would be used for genomic comparisons of lignocellulose degradation systems, which is critical for fungal industrialization study. 

The purpose of the present study was to provide a global understanding through genomic and experimental research about lignocellulosic biomass conversion by *Cerrena unicolor*. This study established the fundamental genomic and genetic resources in *C. unicolor* that can be used as a model for future molecular genetics investigations and breeding. Sequencing of *C. unicolor* SP02 will allow it to be used as a model to understand the regulation of enzyme production in basidiomycete fungi and to provide routes to produce lignocellulolytic enzymes and secondary metabolites at a large scale for industrial applications.

## 2. Materials and Methods

### 2.1. Strain and Cultivation Media

Strain SP02 was obtained from International Joint Laboratory on Microbial Cell Factories of Shandong University of Technology and Georgian National Academy of Sciences. The sample was a native white-rot fungus of Georgia isolated by the Durmishidze Institute of Biochemistry and Biotechnology, Academy of Sciences of Georgia. The strain was grown on Malt Extract Agar (MEA) medium and incubated at temperature 28 °C in the dark. MEA medium (glucose, 3 g·L^−1^; NaNO_3_, 3 g·L^−1^; KH_2_PO_4_, 0.8 g·L^−1^; K_2_HPO_4_, 0.2 g·L^−1^; MgSO_4_·7H_2_O, 0.5 g·L^−1^; yeast extract, 3 g·L^−1^; malt extract, 10 g·L^−1^; agar, 18 g·L^−1^, pH 5.8–6.0) was also used for strain storage and maintenance. Liquid nutrient medium (glucose, 15 g·L^−1^; NaNO_3_, 3 g·L^−1^; KH_2_PO_4_, 0.8 g·L^−1^; K_2_HPO_4_, 0.2 g·L^−1^; MgSO_4_·7H_2_O, 0.5 g·L^−1^; yeast extract, 2 g·L^−1^; pH 5.8–6.0) was used for the preparation of inoculum. 

### 2.2. Isolation of Total DNA and ITS Sequence Analysis 

Strain SP02 was cultivated in the liquid nutrient medium at 28 °C for 5 days. Approximately 200 mg of washed mycelium ground with liquid nitrogen and genomic DNA was isolated using the Plant Genomic DNA Kit (TIAN-GEN, Beijing, China) according to the manufacturer’s instructions. ITS region of genomic DNA was amplified using the highly conserved fungal rDNA gene primers (ITS1 and ITS4), and the sequencing of amplified products was conducted by Genewiz Co., Ltd. (Suzhou, China). The nucleotide sequence has been submitted into the NCBI database. In addition, the ITS gene’s nucleotide sequence was utilized as a query to NCBI to retrieve other relevant sequences, and a homology search was done using the BLAST search method. The neighbor-joining phylogenetic tree was constructed with closely related species using MEGA 6.0 software.

### 2.3. Genome Sequencing, Assembly and Annotation

The obtained DNA of strain SP02 was also subjected to whole-genome sequencing. The genome of strain SP02 was sequenced by the high throughput Illumina HiSeq X-Ten and PacBio Sequel long-read sequencing platforms at Genewiz Co., Ltd. (Suzhou, China). Next-generation sequencing library preparations were constructed following the manufacturer’s protocol. For each sample, 100 ng genomic DNA was randomly fragmented to <500 bp by sonication (Covaris S220, Woburn, MA, USA). The fragments were treated with End Prep Enzyme Mix for end repairing, 5′ Phosphorylation, and dA-tailing in one reaction, followed by a T-A ligation to add adaptors to both ends. Size selection of Adaptor-ligated DNA was performed, and then fragments of ~470 bp (with the approximate insert size of 350 bp) were recovered. Each sample was then amplified by PCR for 8 cycles using P5 and P7 primers, with both primers carrying sequences that can anneal with flowcell to perform bridge PCR and P7 primer carrying a six-base index allowing for multiplexing. The PCR products were cleaned up and validated using an Agilent 2100 Bioanalyzer (Agilent Technologies, Palo Alto, CA, USA), and quantified by Qubit 3.0 Fluorometer (Invitrogen, Carlsbad, CA, USA). Then, libraries with different indices were multiplexed and loaded on an Illumina HiSeq instrument according to the manufacturer’s instructions (Illumina, San Diego, CA, USA). For Illumina, genome sequencing was carried out using a 2 × 150 paired-end (PE) configuration; image analysis and base calling were conducted by the HiSeq Control Software (HCS) + OLB + GAPipeline-1.6 (Illumina) on the HiSeq instrument. For Pacbio, Genomic DNA was sheared, and then 10 Kb (20 Kb if the genome size was larger than 30 M) double-stranded DNA fragments were selected. DNA fragments were end-repaired and ligated with universal hairpin adapters. Subsequent steps were followed as per the manufacturer’s instruction to prepare the SMRTbell library (DNA Template Prep Kit, PACBIO, Menlo Park, CA, USA). 

The library was sequenced in the PacBio Sequel Single-Molecule Real-Time (SMRT) instrument [36]. The low-quality PacBio reads were filtered and assembled to generate circular contig without gaps using HGAP4 4.0/Falcon 0.3 of the WGS-Assembler 8.2 [37,38,39,40,41,42]. Then, the genome was recorrected with software Pilon 1.22 using previous Illumina data or Quiver using Pacbio reads. The Prodigal/Augustus gene-finding software, Prodigal 2.6.3 and Augustus 3.3, were used for finding coding genes [43,44]. Cmscan 1.1.2 was used for ncRNA analysis. Transfer RNAs (tRNAs) were detected in the genome using the program tRNAscan-SE with default parameter settings [45]. rRNA were identified by using RNAmmer [46]. The coding genes were annotated with the NCBI NR database by BLAST. Then the functions of the genes were annotated by the Gene Ontology (GO) database [47], and the pathways were annotated using the Kyoto Encyclopedia of Genes and Genomes (KEGG) database [48]. The carbohydrate-active enzyme analyses of the genome was described by Carbohydrate-Active EnZymes Database (CAZymes/CAZys, http://www.cazy.org/, accessed on 24 February 2021) [49]. The proteins encoded by genes were classified on a phylogenetic classification by using the euKaryotic Orthologous Groups (KOG) of the Clusters of Orthologous Groups (COG) database. Genome overview was created by Circos software to show the annotation information [50].

### 2.4. Growth Characters on Lignocellulosic Materials

#### 2.4.1. Growth Rate and Preference on Substrates 

Lignocellulosic materials corn stalk (CS), wheat straw (WS), rice straw (RS), pine bark (PB), oak bark (OB), peanut shell (PS), and grape seed (GS) were collected from Zibo city, Shandong province. After air drying, the samples were crushed with a pulverizer, and then leached with a 20-mesh sieve to obtain straw powder substrates. Strain SP02 was compared and morphologically observed by using various lignocellulosic materials (substrates) as single carbon sources, respectively. Lignocellulose agar medium (substrates, 15 g·L^−1^; NaNO_3_, 3 g·L^−1^; KH_2_PO_4_, 0.8 g·L^−1^; K_2_HPO_4_, 0.2 g·L^−1^; MgSO_4_·7H_2_O, 0.5 g·L^−1^; agar, 18 g·L^−1^, pH 5.8–6.0) autoclaved at 121 °C for 30 min and medium replaced 15 g·L^−1^ glucose of substrate served as controls. The hypha end of sub-cultured SP02 was cut into 5 mm in diameter and transferred on different plates for incubation at 28 °C under dark conditions. The average fungal growth rate was measured by continuous daily observation on a plate of different carbon sources.

#### 2.4.2. Solid-State Fermentation (SSF)

SSF was performed on agro-waste substrates CS, WS, RS, PB. Seed culture was prepared by inoculating the tested strain in the liquid nutrient medium incubated at 28 °C for 5 days in a shaking incubator. Seed medium was used as inoculum for SSF media. Each flask (150 mL) for SSF, containing 5 g of substrates and 20 mL of culture medium (consisted of KH_2_PO_4_ 0.8 g·L^−1^, K_2_HPO_4_ g·L^−1^, NaNO_3_ 3 g·L^−1^, MgSO_4_·7H_2_O g·L^−1^, yeast extract g·L^−1^), was autoclaved 30 min at 121 °C. Each flask was inoculated by 5 mL seed culture and incubated under shading conditions at 28 °C. The samples of SSF were collected after every 3 days until the 18th for the determination of secreted laccase (Lac), manganese peroxidase (MnP), lignin peroxidase (Lip), CMCase, and FPase (FPA, total cellulase) evaluation [51,52]. Laccase (Lac) activity was determined by oxidation of the 2,2′-azino-bis-(3-ethylbenzthiazoline-6-sulfonate) (ABTS) method [53]. One unit of enzyme activity was defined as the amount of enzyme required to oxidize 1 umoL ABTS/min using an ε420 value for oxidized ABTS of molar absorption coefficients 36,000 M^−1^cm^−1^. MnP activity was measured by oxidation of Mn^2+^ [54]. One unit of enzyme activity was defined as the amount of enzyme required to form 1 umoL of Mn3+/min using an ε240 value for Mn3+ of molar absorption coefficients 6500 M^−1^cm^−1^. Lignin peroxidase (LiP) activity was measured essentially as described by Tien and Kirk [55]. One unit of enzyme activity was defined as the amount of enzyme required to form 1 umoL of veratraldehyde/min using an ε310 value for veratraldehyde of molar absorption coefficients 9300 M^−1^cm^−1^. For CMCase, the reaction mixture contained 1 mL of appropriately diluted enzyme and 3 mL of 1% CMC-Na in 50 mM citric acid buffer (pH 4.8), incubated at 50 °C in water bath for 30 min, and the reaction was terminated by the addition of 3 mL dinitrosalicylic acid (DNS); then the whole mixture was diluted to 25 mL with dH_2_O. Reducing sugar levels in the supernatant was determined at 540 nm [56]. The FPase reaction mixture contained 1 mL crude enzyme and 3 mL of 1 × 6 cm quantitative filter paper in 50 mM citric acid buffer (pH 4.8), and the mixture was incubated at 50 °C for 1 h and measured with the same method of CMCase [57]. CMCase and FPase were calculated as µmoL reducing sugar released per minute per g of compost (U/g). In the SSF study, the same collected samples were used to perform at least two parallel analyses and all enzymatic reactions were performed in triplicate. On the last day of cultivation (day 18), different cultures were processed for component and physical change analysis [58]. Scanning electron microscopy was used to observe and analyze physical changes by a microbe in treated substrates. Images of the substrates were taken using a Thermo Scientific Apreo scanning electron microscope (SEM), belonging to scientific and technical services center of the Shandong University of Technology.

### 2.5. Data Availability and Accession Numbers

The genome sequencing data and annotation of *C. unicolor* SP02 results in this paper are associated with NCBI BioProject: PRJNA704632 and BioSample: SAMN18051415. Raw sequencing data have been deposited in the NCBI Sequence Read Archive (http://www.ncbi.nlm.nih.gov/sra, accessed on 24 February 2021) under accession no. SRR13780188.

## 3. Results and Discussion

### 3.1. Phylogenetic Analysis of Strain SP02

**Figure 1:**
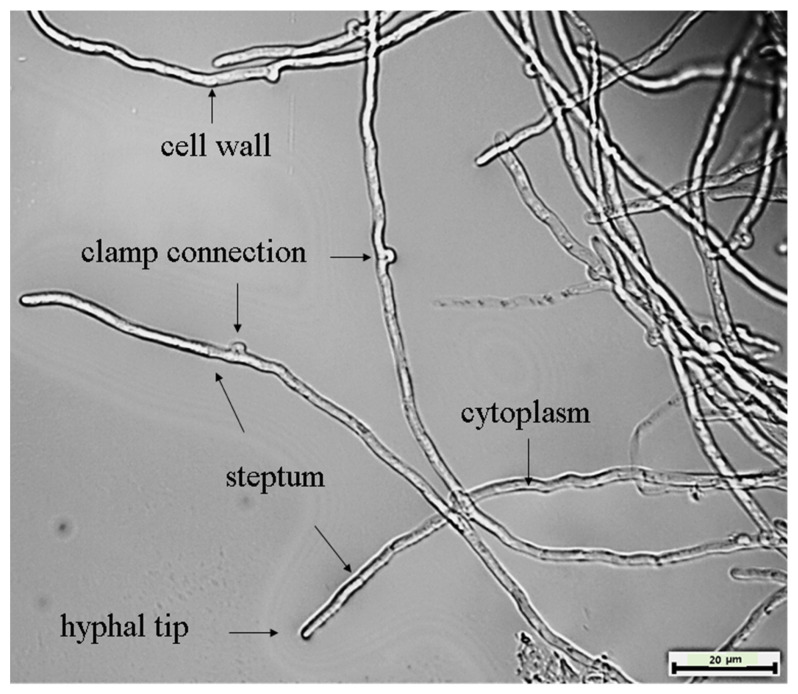
Cellular morphology of SP02 hypha (magnification 10 × 100) from liquid nutrient medium inoculated SP02 at 28 °C for 5 days.

**Figure 2:**
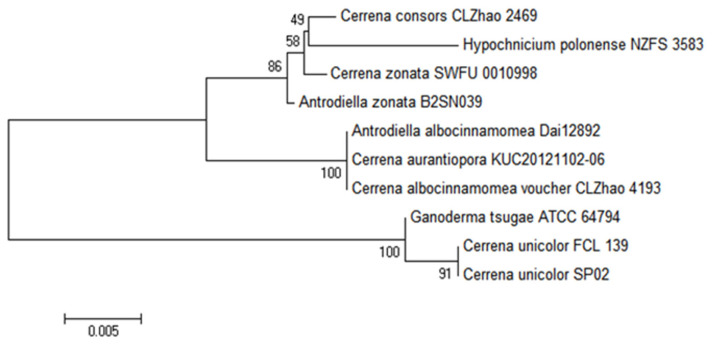
Neighbor-joining phylogenetic tree of SP02 and related members based on ITS sequences blast. The phylogenetic tree was constructed using the MEGA 6.0 program. Bootstrap values (expressed as percentages of 1000 replications) >50% are indicated at the branch points. The scale bar indicates 0.005 nucleotide substitutions per site.

The fungus *C. unicolor*, often known as a mossy maze polypore, is a wood-degrading basidiomycete of the Polyporaceae family that causes extensive white rot [59]. The selected isolate coded as SP02 was identified based on its morphology and the ITS-5.8S ribosomal gene sequence method. From the morphological study, it was observed that the isolate developed a pure white mat with typical concentric zones in Petri dish cultures. Under microscopic examination, the trimitic hyphal system that produces single basidiospores in its fruiting body was also observed. The micro characteristics of the mycelium structure of this fungus have been shown in Figure 1. The phylogenetic analysis of strain SP02 based on ITS sequences was conducted by MEGA 6.0 with related species in Figure 2 to show the phylogenetic relationships. ITS sequence of strain SP02 was submitted to NCBI under the accession number: MW883617. 

The phylogenetic tree grouped strain SP02 with *C. unicolor*, *C. cosors*, *C. zonata*, *C. aurantiopora, C. albocinnamomea*, etc., by using the neighbor-joining method. ITS blast of the strain SP02 showed a 100% similarity with *C. unicolor* FCL 139 (DQ056858.1). Strain SP02 was successfully clustered to *C. unicolor* and closest to the strain *C. unicolor* FCL 139, which is reported as an extensive white-rot wood-degrading basidiomycete [22,60]. The morphological characterization and phylogenomic results are consistent with the consensus taxonomic status of *C. unicolor* and suggested that SP02 is a member of Cerrena. Based on these results, the strain SP02 was identified as *C. unicolor* SP02.

### 3.2. General Genome Characteristics of C. unicolor SP02

**Figure 3:**
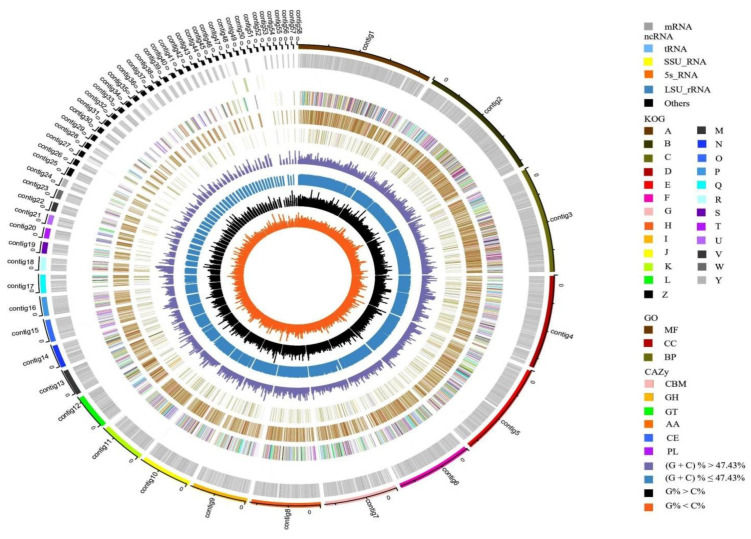
Circular genome map of *C. unicolor* SP02. From the outside to the center: circle 1: genome contigs; circle 2: mRNA; circle 3: ncRNA; circles 4 to 6: the predicted protein-coding genes by using KOG, GO, and CAZy databases, respectively, where different colors represent different function classifications; circle 7: (G + C) % > 47.43%; circle 8: (G + C) % ≤ 47.43%; circle 9: G% > C%; circle 10 G% < C%.

Genome sequencing yielded a total of 34.8 million pairs of reads and 5220 Mb bases in Illumina HiSeq X Ten Pass Filter Data and was assembled with a combination of PacBio Sequel data. General genome characteristics of *C. unicolor* SP02 are shown in Table 1 and Figure 3. The final genome assembly resulted in a total length of 42.8 Mb and the GC content was 47.43%. The complete genome was composed of 58 sequences, in which the longest contig length was 4.37 Mb and the shortest contig length was 33.8 Kb. The N50 of the assembly was 2.48 Mb. A total of 12,277 protein-coding gene sequences were predicted, with an average CDS length of 1716.20 bp. The genome was found to have 305 Non-coding RNA that contain 230 tRNA, 49 rRNA, and 26 other ncRNA genes.

Repeat region analysis reports 1533 retroelements in the *C. unicolor* SP02 genome, the majority of which belonged to the LTR (long terminal repeat) family (1076), and the others were 437 LINEs (long interspersed nuclear elements) and 20 SINEs (short interspersed elements). There were also 166 DNA transposons, 2214 unclassified repeats, 3220 simple repeats, and 631 low-complexity repeats detected in the genome (Supplementary Tables S1–S5).

### 3.3. Gene Function of C. unicolor SP02

Annotation and functional classification results of 12,277 genes were obtained based on five datasets of the NR database, COG/KOG database, KEGG database, GO database, and CAZys database. A total of 10,470 (85.28%) were annotated in the NR database, followed by the KEGG database (6386, 52.02%), GO database (5904, 48.09%), COG/KOG database (5595, 45.57%), and CAZys database (1193, 9.72%). 

**Figure 4:**
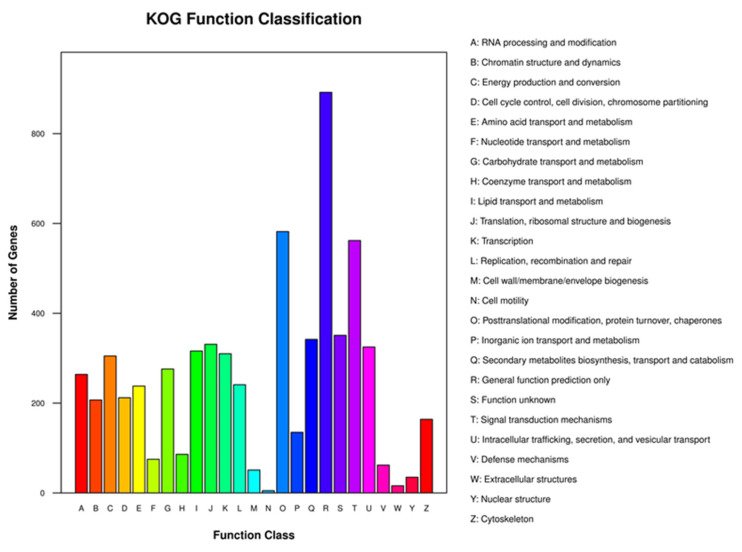
Clusters of the KOG functional classification of *C. unicolor* SP02 genes. A total of 5595 genes were assigned to 25 classifications. The capital letters on the x-axis indicate the KOG categories as listed on the right of the histogram.

The KOG classification of proteins annotation results showed that a total of 5595 genes were annotated in 2940 KOG categories and assigned to 25 clusters of KOG classifications. As Figure 4 shows, (R) General function prediction of only functional categories contained the largest number of genes (892). The top of the gene-rich KOG categories were associated with function class: (O) Posttranslational modification, protein turnover, and chaperones (582); (T) Signal transduction mechanisms (562); (S) Function unknown (351); (Q) Secondary metabolites biosynthesis, transport, and catabolism (342); (J) Translation, ribosomal structure and biogenesis (331). Moreover, the function classes “Intracellular trafficking, secretion, and vesicular transport” and “Carbohydrate transport and metabolism” involved 276 and 325 genes, respectively. They may be related to the lignocellulosic deposing function of *C. unicolor* SP02. Secreted enzymes like lignin peroxidase have been known as an important extracellular enzyme to breaks down lignocellulose [12]. Carbohydrate transport and metabolism function are essential in polysaccharides and lignin degradation, which can catalyze the transport of multiple substrates including ions, carbohydrates, lipids, amino acids, peptides, nucleosides, and other small molecules [61]. 

**Figure 5:**
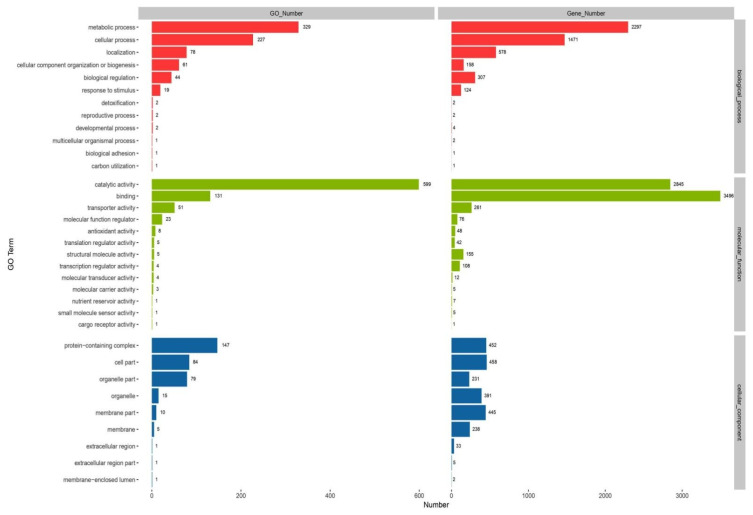
The Gene Ontology (GO) function annotation of *C. unicolor* SP02.

GO functionally annotated 5904 protein-coding genes of *C. unicolor* SP02, in which most predicted proteins have more than one GO term. These genes were functionally annotated with 674 GO terms to understand the major biological and molecular role of the predicted function. Genes were categorized by biological process, cellular component, and molecular function categories. The molecular function class was largest, followed by the biological process and cellular component. As Figure 5 shows, genes were assigned to different GO classes. The genes were mainly assigned to “cell part” (485 genes & 84 GO terms), “protein-containing complex” (452 genes & 147 GO terms), and “membrane part” (445 genes & 79 GO terms) in the “cellular component” category. In the “biological process” category, “metabolic process” (2297 genes & 329 GO terms), “cellular process” (1471 genes & 227 GO terms), and “localization” (578 genes & 78 GO terms) contained most genes, and the two highest classes in “molecular function” category were “binding” (3496 genes & 599 GO terms) and “catalytic” (2845 genes & 131 GO terms).

The “Biological process” GO category revealed strong representation (>200 genes) of the “oxidation-reduction process” (GO:0055114), “transmembrane transport” (GO:0055085), “protein phosphorylation” (GO:0006468), and “DNA integration” (GO:0015074). The “Cellular component” GO category revealed the stronger occurrence of GO terms corresponding (>200 genes) to “integral component of membrane” (GO:0016021), “nuclear pore” (GO:0005643), and “membrane” (GO:0016020). The “Molecular function” category revealed stronger occurrence (>400 genes) of GO terms corresponding to “protein binding” (GO:0005515), “nucleic acid binding” (GO:0003676), “ATP binding” (GO:0005524), and catalytic activity (GO: 0003824). Moreover, classes were also found relevant to lignocellulosic degradation like “cellulase activity” (GO:0008810), “peroxidase activity” (GO:0004601), and “aromatase activity” (GO:0070330). 

**Figure 6:**
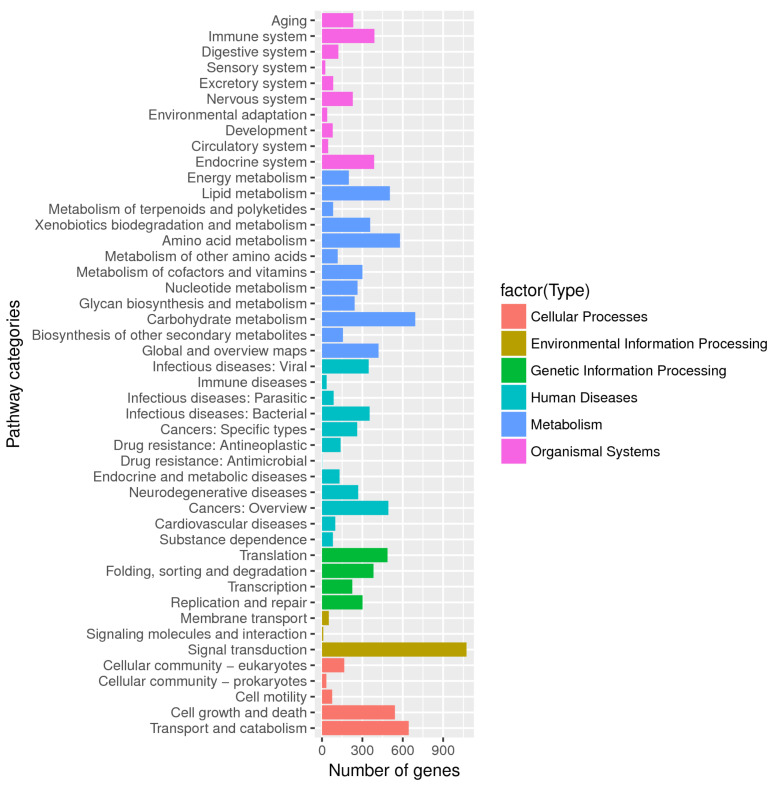
The KEGG function annotation of *C. unicolor* SP02. Distribution of genes in different KEGG categories.

Approximately 6386 genes were successfully annotated in the KEGG database. Twelve metabolism categories in KEGG were highly enriched as Figure 6, as shown, including carbohydrate metabolism (693), amino acid metabolism (579), lipid metabolism (504), global and overview maps (420), and xenobiotics biodegradation and metabolism (358). Carbohydrate metabolism provides fungi benefits by take up and they utilize carbon from plants in ectomycorrhizal symbiosis [62]. *C. unicolor* SP02 contained genes rich in “Signal transduction” (1074) under “environmental information processing,” which regulates the production of extracellular enzymes and plays a role in mediating the lignin substrate specificity [63]. “Membrane transport” and “endocrine system” are related to the secretion and transportation of extracellular enzymes. “Transport and catabolism” and “xenobiotics biodegradation and metabolism” are related to the ability of lignin degradation and detoxification. A total of 3856 of annotated genes were assigned to the orthologs of 379 KEGG pathways, with most genes having multiple pathways, and among them, a large number of genes were involved in the KEGG Orthologys associated with lignocellulosic decomposing: Starch and sucrose metabolism (ko00500), Degradation of aromatic compounds (ko01220), Phenylpropanoid biosynthesis (ko00940), Dioxin degradation (ko00621), Benzoate degradation (ko00362), Phenylalanine metabolism (ko00360), which were involved in degradation and metabolizing of phenylalanine, styrene, geraniol, chlorocyclohexane and chlorobenzene, fluorobenzoate, dioxin, xylene, toluene, aminobenzoate, limonene and pinene, aromatic compound, polycyclic aromatic hydrocarbon, and bisphenol.

**Figure 7:**
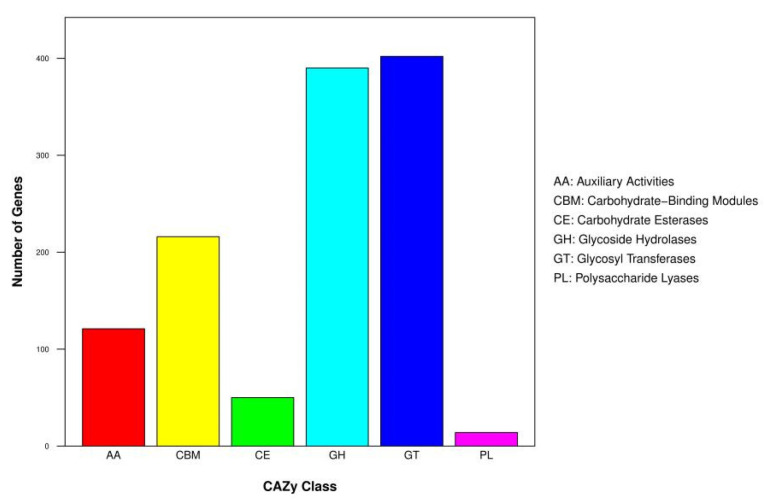
Distributions of CAZymes gene families in *C. unicolor* SP02 genome.

A total of 1193 CAZyme-coding genes of SP02 were annotated, included 402 Glycosyl Transferases (GTs), 390 Glycoside Hydrolases (GHs), 216 Carbohydrate-Binding Modules (CBMs), 121 Auxiliary Activities (AAs), 50 Carbohydrate Esterases (CEs), and 14 Polysaccharide Lyases (PLs), which accounted for 33.70%, 32.69%, 18.11%, 10.14%, 4.19%, and 1.17%, respectively (Figure 7). To degrade the polysaccharides, wood decay fungi secrete a variety of GHs and CEs classified into various sequence-based CAZys families and their appended CBMs. In various CAZys, GHs are the most diverse group of enzymes in the degradation of biomass. A lot of GHs families have been classified to date [64]. Many of them are responsible for the hydrolysis of the carbon-oxygen-carbon bonds that link the sugar residues in cellulose and hemicelluloses [65,66]. Among those likely involved in cellulose degradation, genes of GH1, GH3, GH5, GH6, GH7, GH12, GH45, and GH55 were represented and identified. To digest hemicellulose, GH3, GH10, GH12, GH43, GH51, and GH79 were assigned. Plant pathogenic, hemibiotrophic, and necrotrophic fungi generally contain more GH1 degrading enzymes than biotrophic fungi, which have almost none [67]. The genome of SP02 encodes five GH1 genes, indicating that it tends to a necrotrophic lifestyle. Rich CBM1 genes were engaged to help SP02 attach mainly to crystalline cellulose and may serve to concentrate enzymes on cellulose surfaces [68]. Esterases of SP02 were mostly distributed in the CE4, CE6, CE8, CE11, CE12, and CE16 classes. These CEs help the O-de-N-deacylation of acetylated glycosyl residues in hemicellulose, pectin, and lignin units of the plant [69]. GH 29 fucosidases and pectate lyases belong to PL1 and PL3 possessed in SP02 suggested that the fungi probability have the ability of weak parasitism associate with the occasional observation of *C. unicolor* on alive trees [60]. In the classification of the CAZymes database, lignin-degrading enzymes were subdivided into the AA classes [12]. CAZyme results showed that SP02 possesses a large number of AAs, including lignin-oxidizing enzymes AA1, AA2, AA3 classes; lignin-degrading auxiliary enzymes AA4, AA5, AA6 classes; polysaccharide decomposing related AA7, AA9, AA10 classes; and pyrroloquinoline quinone-dependent oxidoreductase class AA12. These CAZymes represent specific functions and colonize the ability of various plant substrates. Most predicted CAZyme-coding genes supporting the saprophytism lifestyle of SP02 by using lignocellulosic sources indicate that SP02 maintains great enzymatic diversity supporting lignocellulose attack, which is probably based on polysaccharide degradation and oxidation-reduction activity.

### 3.4. Genes Related to Biodegradation in C. unicolor SP02

#### 3.4.1. Lignin-Degrading Enzymes

*C. unicolor* SP02 often grows on dead hardwood, which consists of cellulose, hemicellulose and lignin. Saprophytes need to secrete an array of lignin-degrading enzymes to break down recalcitrant lignin in the plant cell wall [70]. Unlike the hydrolysis of polysaccharides, lignin digestion is considered an enzymatic combustion process. The oxidation of a variety of lignin-related compounds including aromatic pollutants responding with ligninolytic enzymes, peroxidase synergizing the redox condition, and the Fenton reaction to maximize aromatic polymer degradation [71,72]. Gene results show lignin-degrading enzyme systems of SP02 involving a series of oxidoreductases. Lignin-oxidizing enzymes mainly include laccase (EC 1.10.3.2), lignin peroxidase (EC 1.11.1.14), manganese peroxidase (EC 1.11.1.13), and cellobiose dehydrogenase (CDH, EC 1.1.99.18). Lignin-degrading auxiliary enzymes majorly include hydrogen peroxide-generating enzymes such as glyoxal/methylglyoxal oxidase (GLX, EC 1.2.3.15), pyranose oxidase (PDC, EC 4.1.1.1), vanillyl-alcohol oxidase (EC 1.1.3.13), and benzoquinone reductases (EC 1.6.5.-). Apart from these ligninolytic enzymes, aromatic compound-degrading, detoxifying enzymes, and highly reactive free radicals such as hydrogen peroxide, hydroxy radicals, superoxide, and reactive singlet oxygen ions also play a crucial role in the degradation of lignocellulosic units, in which superoxide dismutase (SOD), heme-peroxidases, dye-decolorizing peroxidase, alcohol dehydrogenase, iron reductase, ferredoxin, catalase, oxidoreductase, and another large set of enzymes involved in Flavin-containing Monooxygenases (FMOs) and Cytochrome P450s (CYPs) families were found in the SP02 genome. FMOs are importantly recognized when applied in the metabolism of nitrogen-, sulfur-, and other nucleophilic heteroatom-containing xenobiotics [73,74]. CYPs play diverse and critical roles in metabolism and fungal adaptation to specific ecological niches [75], are involved in the breakdown of various xenobiotics and lignin metabolites released by the action of extracellular peroxidases [76,77], and assist in stereo- and regio-specific oxidation of substrates [78]. 

A total of 165 CYP genes in the SP02 genome were annotated and 149 of them were classified into 30 subfamilies. In comparison, this number is close to those such as *Trametes trogii* (158) and *Phanerochaete chrysosporium* (152), and far higher than *Agaricus bisporus* (109) [79,80]. NADPH-cytochrome P450 reductase, cytochrome b5, and cytochrome b5 reductase were also identified in SP02 genome as potential cytochrome P450 NADPH redox partners. The SP02 genome was also coded other enzymes related to the lignocellulosic degrading process, such as glutathione S-transferase (GST, EC 2.5.1.18), and it was found that it cleaves the β-aryl ether (β-O-4) bond, the most common bond between aromatic subunits in lignin [81]. Alcohol oxidase and PDC reacted with abundant alcohol and glucose to generate reactive oxygen, which could, in turn, be used by peroxidase for lignin depolymerization [82]. PDC assists white-rot fungus and produces ethanol directly from lignocellulose; modification on PDC is the key to construct industrial strains of lignin-degrading ability to producing xylitol, lactic acid, or pyruvate directly [83,84]. CDH generates hydroxyl radicals for the Fenton reaction and also functions synergistically with manganese peroxidase in lignin degradation [85]. Besides, SP02 codes lytic polysaccharide monooxygenase (LPMO), catechol 1,2-dioxygenase, ornithine carbamoyltransferase (OTC), carboxymethylenebutenolidase, pentachlorophenol monooxygenase, phenol 2-monooxygenase quinone reductase, and unspecific peroxygenase related to lignocellulosic degradation. These results suggest that the strain SP02 possesses a complicated lignin metabolism system.

#### 3.4.2. Cellulose Degrading Enzymes

Cellulose is the most abundant component in plant cell walls, made by the polymerization of glucose units that results in the formation of a microfibril framework for other components to join [86]. SP02 have mainly classical cellulases include endo-β-1,4-glucanases (EG, EC 3.2.1.4), exo-β-1,4-glucanase (CBH, EC 3.2.1.91), and β-glucosidase (BGL, EC 3.2.1.21) for the hydrolysis of cellulose. Endo- and exo-β-1,4-glucanases hydrolases cellulose chains by releasing gluco-oligosaccharides while exo-β-1,4-glucanases and β-glucosidases liberate cellobiose from end chains of cellulose. SP02 CBHs contain both CBH1 and CBH2, which cleave at different sites on reducing or non-reducing ends of the cellulose. BGLs support SP02 to release individual glucose units from the shorter oligosaccharide chains. Along with classical cellulases, strong oxidoreductases such as CDH and LPMO also participate in the degradation of cellulose. CDH and LPMO enzymes work cooperatively for depolymerizing cellulose, as CDH produces highly reactive hydroxy radicals through Fenton’s chemistry, which plays a dual role by modifying lignin and providing electrons for LPMO-based cellulose degradation [87]. Physiological connections exist between aldose 1-epimerase (AEP, EC 5.1.3.3), CDH, and cellulases, and AEPs of SP02 may interact with CDH via generation of the preferred CDH substrate cellobiose β-anomer [68]. Moreover, exo-1,3-β-glucanase (EC 3.2.1.58), endo-1,3-α-glucanase (EC 3.2.1.59), glucan endo-1,6-β-glucosidase (EC 3.2.1.75), α-glucosidase (EC:3.2.1.20), α-amylase (EC:3.2.1.1), and pectate lyase (EC:4.2.2.2) were found involved the cellulose hydrolyzation in the complete genome of SP02.

#### 3.4.3. Hemicellulose-Degrading Enzymes

Fungal degradation of hemicellulose is performed by a specific set of CAZymes; xylan hydrolysis into xylobiose releases D-xylose units from xylooligosaccharides and hydrolyses xylobiose units to monomeric units, respectively [88]. SP02 containing a series of genes coding xyloglucan-specific endo-β-1,4-glucanase (EC:3.2.1.151), xyloglucan-specific exo-β-1,4-glucanase (EC:3.2.1.155), endo-1,4-β-xylanase (EC 3.2.1.8), xylan 1,4-β-xylosidase (EC 3.2.1.37), α-L-arabinofuranosidase (EC 3.2.1.55), arabinan endo-1,5-α-L-arabinosidase (EC 3.2.1.99), and putative laminarinase relate to the hemicellulose degradation steps [89]. Hemicelluloses include various types of xyloglucan, glucomannan, mannan, xylan, arabinoxylan, and arabinogalactan. In hardwood decay, mannan endo-1,4-β-mannosidase (EC 3.2.1.78), β-mannosidase (EC 3.2.1.25), α-galactosidase (EC 3.2.1.22), and β-galactosidase (EC 3.2.1.23) play more important roles as well [69]. β-glucuronidase (EC 3.2.1.31) is involved in hydrolyzing beta-glucuronosyl and 4-O-methyl-beta-glucuronosyl residues of arabinogalactan-proteins (AGPs) processing [90]. Acetylesterase (EC 3.1.1.6), feruloyl esterase (EC 3.1.1.73) and acetyl xylan esterase (EC 3.1.1.72) are involved in the acetyl O-de-N-deacylations. Cellulases, CDH, and LPMOs decomposing cellulose are also involved in the hydrolysis of xyloglucan and β-glucan backbone structures [91,92]. 

#### 3.4.4. Other Degrading Enzymes

Pectin is a non-cellulosic polysaccharide component of the plant cell wall, and it is majorly comprised of galacturonic acid, which is intricately connected with the cellulose and hemicellulose units. SP02 coding an arsenal of enzymes involved in depolymerization of pectins such as endo-polygalacturonase (EC:3.2.1.15), exo-polygalacturonase (EC:3.2.1.67), rhamnogalacturonan endolyase (EC:4.2.2.23), α-L-rhamnosidase (EC:3.2.1.40), pectate lyase (EC:4.2.2.2), pectin esterase (EC:3.1.1.11), and rhamnogalacturonan acetylesterase (EC:3.1.1.86). Endo- and exo- polygalacturonases act by cleaving linkages of homogalacturonan to release D-galacturonic acid [93]. Rhamnogalacturonan endolyase and α-L-rhamnosidase are involved in depolymerization of rhamnogalacturonan [70]. Esterases join in the acetyl removal in pectin degradation. Besides, SP02 is particularly rich in genes that catalyze the decomposition of chitin, containing 49 and 7 genes assignable to GH18 and GH19 chitinases, respectively. Lysozyme is known for microbial cell wall degradation, and chondroitin AC lyase is important in algicidal fungi caused cyanobacterial cells elimination [94]. Gene precoding these enzymes may support SP02 predominantly in the wild saprophyte community, and help its superiority in saprophytic nutrition competition. 

### 3.5. Cultural Characteristics 

#### 3.5.1. Growth of *C. unicolor* SP02 on Agro-Wastes

**Figure 8:**
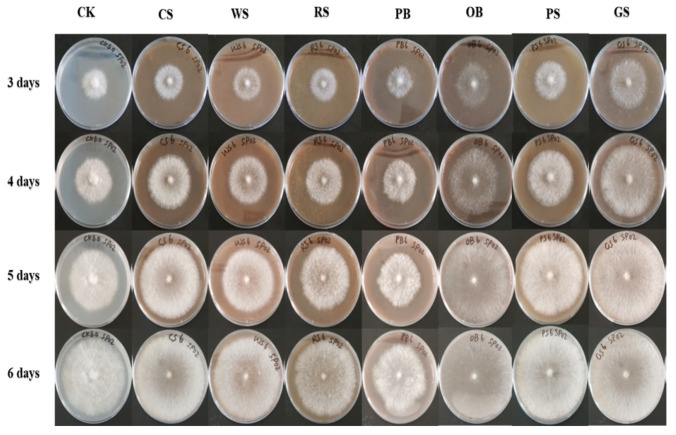
Cultural characteristics of *C. unicolor* SP02 on plates of different single carbon source. CK: glucose; CS: corn stalk; WS: wheat straw; RS: rice straw; PS: peanut shell; CH: cottonseed husk; PB: pine bark; OB: oak bark; GS: grape seed.

Data in Figure 8 record the growth of *C. unicolor* SP02 colony on a plate by using different agro-waste substrates as a single carbon source. SP02 appeared to use all of the tested agro-industrial waste. Early development of SP02 is commonly characterized by initial diffuse growth and branching of individual hyphae, which then resolve into cords as the growing front moves outward. The colony size and number of hypha links increase through time. Then with fast growth, the colony center is characterized by selective loss of connections and thinning out of the fine mycelium and weaker cords that give rise to a decrease in the network density with increasing colony area on the lignocellulosic carbon source. A sparse branching tree-like structure forms in the peripheral growth zone from tip growth and sub-apical branching. With the growth colony, edges become denser and full of overlapping hyphae. By contrast, the colony on control (glucose) kept dense mycelium around the colony center. In plates of lignocellulosic carbon source, solid substrate presented at the bottom of medium showed no direct contact with healthy developed hypha. Close observation obtained a clear circle around the edge of the colony, which is common in the ligninolytic product process and explained as a higher amount production of secondary metabolites [95]. In this case, the fading color of substrates wrapped in agar means white-rot affection of SP02 still exist under these nutritional condition.

In qualitative estimations, colony diameter was recorded in continued observation. Growth rate of SP02 from highest to the lowest were: grape seed (15.44 ± 0.38 mm/d), oak bark (14.98 ± 0.33 mm/d), peanut shell (13.40 ± 0.38 mm/d), corn stalk (13.12 ± 0.38 mm/d), wheat straw (11.98 ± 0.40 mm/d), control (11.68 ± 0.53 mm/d), rice straw (11.00 ± 0.35 mm/d), pine bark (9.45 ± 0.54 mm/d). Colony size was recorded higher in most natural substrate medium than control with the same incubating time (except rice straw and pine bark). The most promising substrate seemed to be corn stalk and peanut shell, which combine, characterized with fast formed health and dense mycelium. As a comparison, other substrates resulting in problems such as sparse mycelium and relatively slow growth.

The growth *C. unicolor* SP02 is also tested on cellulose by use sodium carboxymethylcellulose and avicel cellulose as a single carbon source and result in the arrest of growth (not shown in figures). *C. unicolor* SP02 was found to prefer natural plant-derived carbon sources over pure chemical ones, with the preference were lignocellulose > glucose > cellulose. Given grape seed and pine barks are the lignin-led (lignin components > 53%) substrate in tested materials, it could be speculated that natural substrates provide essential nutrients to promote the growth of SP02 by continually offering growth active molecules, which comes from a soluble metabolite of lignin and soluble suppressor and promote factors make the difference. In general, glucose is known for the optimum production of fungal biomass and enzymes [96]. In most cases, it is hard to create an effective adjustment strategy on extracellular enzymes in nutritional deficiencies, as complex molecules are not easily utilized compared to simple monosaccharides like glucose [97]. But it is contradicted that SP02 preferred the complex plant-derived substrates while they were difficult for other fungi to utilize. SP02 seems well adaptable to fungi, which showed fast growth on some substrates of hard use for white-rot fungi *Pycnoporus cinnabarinus* [98]. It can be explained with the capability of some fungal species of the utilization of different carbon sources and diversified enzyme synthesis has already been proposed as a mechanism of slow adaptation of higher white-rot fungi to changing environmental factors [99,100]. 

#### 3.5.2. Lignocellulolytic SSF Characters

Solid state fermentation often offers more advantages in the production of enzymes than submerged techniques using agro-industrial waste as a carbon source [101,102]. In this experiment the enzymes including Lac, LiP, MnP, CMCase and FPA were analyzed in *C. unicolor* SP02 culture grown under SSF using different agriculture wastes. As shown in Table 2, the expression of Lac, CMCase, FPA activities of SP02 were found in all experimental substrates, it was also observed that the MnP and Lip showed variation in production on different substrates. SP02 could keep a long time MnP production at the early stage of fermentation on corn stalk and wheat straw, but it appeared relatively late on rice straw, and hardly secreted in pine bark. Lip of SP02 appeared early in field straws, especially in corn stalks. In pine bark incubation, SP02 spends a longer time in Lip production. It seems to correspond with the hysteresis effect of SP02 mycelium growth on rice straw and pine bark plate in Figure 8 that the irregular edge style of RS/PB incubated SP02 colonies are obvious differences from the neat edge colony style of other substrate incubated ones. Lac of SP02 in RS increased substantially in the late period show off-growth condition may cause stimulation of metabolite production to produce higher amounts of secondary metabolites under suboptimal conditions. It happened occasionally that adverse conditions to a white-rot fungal cell instead have positive effects on lac expression, which can be attributed to the strategies possessed by the organisms for their survival under extreme environments [103,104,105]. Based on this, we speculate inhibiting compounds and protective factors exist and antagonization in RS/PB cultivation, which forced SP02 to change the metabolic activity of lignocellulosic secretases to maintain steady growth. These lifestyles lean on the different strategies of SP02 to degrade widely diverse plant biomass. Hence, the carbohydrate degrading enzymes toolkit may be relevant to explain the lifestyle diversity and materials range. 

Lac active showed the greatest difference on substrates. A comparison of the highest production in Table 3, corn stalk got the highest amount of 177.26 U/g at day 9, followed by rice straw expressed 113.36 U/g at day 12. The laccase activities obtained on corn stalk and rice straw in the present study are much higher than those on wheat bran, which only reached 15.88 U/g on day 15. The lac peak expression of SP02 in wheat straw is similar to reported another *C. unicolor* strain in wheat bran [106]. With the same effect, bulky low-cost straw may be preferable substrates for biodegradation. Bran and straw can induce and increase the natural mediators and enhanced the rate of laccase-catalyzed oxidation to improve biodegradability [107]. Bran from wheat/rise is a suitable substrate for industrial Lac production that induced Lac activities is reported much higher than various agro-industrial waste residues substrates [108,109,110]. In the present study, straw from corn and rise can improve laccase-producing of SP02. The inductive laccase capability of the substrate directly relates to its phenolic compound content and the cellulose content of substrate could also act as an activator of laccase activity [111,112]. Some substrates provides fungi with an environment close to their natural habitat, with which the fungus would probably be more stimulated for the secretion of lignin-degrading enzymes [113]. *C. unicolor* is reportedly found on woods of genus Aesculus, Fraxinus, Acer, Betula, Fagus, or Quercus, but is very rarely reported on conifers [1], thus pine bark is far from the natural habitat of *C. unicolor* that may make trouble for SP02 to adaptation. SP02 is a good producer of MnP in corn stalk cultivation that the highest activity was revealed 7.04 U/g, followed by 6.53 U/g in rice straw. The CMCase of SP02 produced well in wheat straw 7.88 U/g, followed by 7.14 U/g and 6.10 U/g in corn stalk and rice straw respectively. While as other enzymes are produced at very low levels. It is the same with early reports that showed the waste materials in SSF are the effective way for Lac and MnP enzyme production from *C. unicolor* [105,114].

As Table 4 showed, SP02 bring maximum consumption of lignocellulosic compounds in corn stalk, which caused above half weight losses of lignin and hemicellulose in 18 days’ SSF. The same with corn stalk incubation, lignin and hemicellulose consumption was much higher than cellulose in wheat straw. But in rice straw, cellulose loss was higher than lignin and hemicellulose. All the compounds were hard to bioconversion in pine barks. There was little difference between corn stalk, wheat straw and rice straw on total weight loss. The disparity between the total weight and main polymers consumptions indicates SP02 efficiency bioconverse insoluble high polymers into soluble molecules and oligomers and provides SP02 a nutritious environment. In the bioprogress, SP02 modified substrates from resistant materials. 

**Figure 9:**
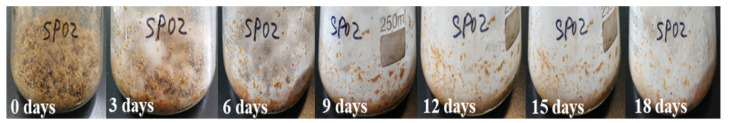
Cultural characteristics of *C. unicolor* SP02 in corn stalk cultivation.

The substrates grain of plant particles decreased in size after the treatment with strain SP02 in SSF. The variation in appearances of substrate grain was also observed as shown in Figure 9. The change in appearances and size is due to consumption of corn stalk as discussed in Table 4. The decolorization of corn stalk materials suggested that efficiency of lignolytic oxidize system of SP02 decreased chromophoric groups such as carbonyl, quinone and phenol hydroxyl in lignin of lignocellulose. Reduction of particle size, porosity, lignin structural disturbance as well as cellulose access, crystallinity, polymerization degree, hemicellulose shielding and cellulose fibers packaging were all emphasized as parameters affecting digestibility of varying degrees and in various hydrolyte process stages [115,116,117].

**Figure 10:**
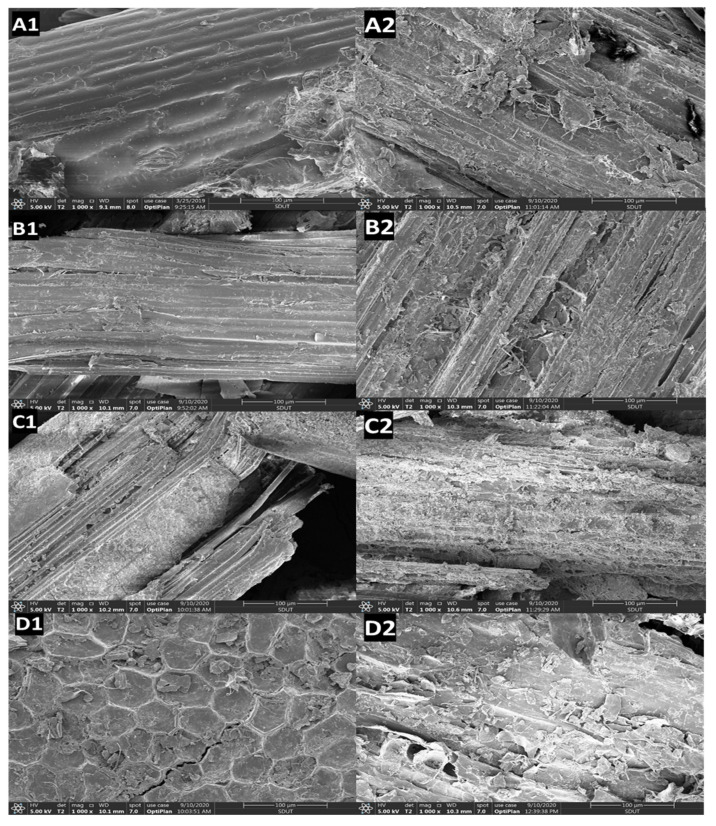
SEM images of SSF cultures: (**A1**) untreated corn stalk; (**A2**) *C. unicolor* SP02 treated corn stalk; (**B1**) untreated wheat straw; (**B2**) *C. unicolor* SP02 treated wheat straw; (**C1**) *C. unicolor* SP02 treated rice straw; (**C2**) *C. unicolor* SP02 treated rice straw; (**D1**) untreated pine bark; (**D2**) *C. unicolor* SP02 treated pine bark.

Morphological examination of the bio-treated substrates is essential because the source of the bio-based hydrolysis technique has a large impact on the dimension and properties of the modified substrates. Thus, SEM analyses were performed to investigate the morphology of different SSF cultures. SEM images of the untreated and treated substrates with *C. unicolor* SP02 gave evidence of the physical changes that occurred during the treatment as shown in Figure 10. The morphology of the untreated substrates varied among different species. Cornstalk, wheat straw and rice straw are mainly agro-waste of gramineous field crops, these substrates consist of mostly stalks and small quantities of leaves, enriched vascular tissue, which was observed in a parallel arranged arrangement regularly and bound firmly in the fiber direction (Figure 10(A1–C1)). Pine bark is mainly cascading arranged empty cavity periderm cork cell, which kept a lot of suberized cell wall filled the air (Figure 10(D1)). These untreated substrates had compact fibrillar structures that appear smooth and the ordered arrangements can be observed on the surface (Figure 10(A1–D1)). The major improvement was observed after bio-pretreatments. SEM images of substrates revealed cuticle waxy layer appeared to be almost desquamated and the component partly exfoliated from the outside epidermis. The microfibers in the cell wall structure were completely disrupted and formed a new pattern with an expanded surface area. It appears that some bio-pretreatment generated a more conglomerate texture with a sponge-like structure. SP02 grows through the breaks of natural biomass structure and grows cobwebby inside cracks (Figure 10(A2–C2)). Bio-incising effect is much more visible on treated substrates, major structure did not show much change after SSF, hypha proliferation put up breaks and the sharp edge being passivated. The phenomenon more obvious on field crops waste in these treated substrates. Three treated field substrates showed disrupted surfaces and flaking out. The contact with the lignocellulolytic enzyme caused the surface to roughened, formed densely corroded marks or holes. The external fibers are loosened after fungal treatment. On treated materials, microorganisms erode seriously, most the surface erosion has perforated. Scan of recognizable transfer passage tissue on substrates shows SP02 decay natural lignocellulosic biomass by mainly effect on the amorphous area without destroying the principal fiber structure, indicate the bio-modify process kept most cellulose binding. The bio-corrosion is relatively slight on pine barks (Figure 10(D2)), for the substrate lack structure for mycelial attaches and access. Generally, enzymes absorb on the surface of cellulose fibers and hardly enter the inner of cellulose fibers because enzymes are larger than the capillaries in the primary cell wall of cellulosic substrates [118]. 

The surface of biotreated substrates was loose and rugged and exposed more porous internal surface, which means SP02 increased the surface area of substrates and made the internal sites to be more available for the next stage of enzymatic hydrolysis. The fungal hydrolysis of cellulose is a surface-dominated phenomenon that need direct contact with the substrate. The surface area is generally considered as an important role in the accessibility and adsorption of enzymes, and the enzymatic hydrolysis efficiency is limited by the accessible surface area [119,120]. Biotreatment solubilizes the lignin and hemicelluloses and thereby disrupts the lignocellulosic composite material linked by covalent bonds, hydrogen bonds, and van der Waals forces, which make cellulose more accessible to enzymes [121]. The high available surface area of SP02 treated lignocellulose allows easy enzyme penetration, absorption and lignocellulosic material conversion to monosaccharides. 

## 4. Conclusions

The versatile biopolymer degradation potential and the pharmaceutical potential render *C. unicolor* SP02 an interesting model organism for bioconversion, degradation, and enzymes production studies. In this study, we performed de novo sequencing and assembly of *C. unicolor* SP02 genome. This is the first de novo assembly and annotation of a *C. unicolor* genome by using the combination of Illumina Hiseq X Ten and the PacBio Sequel sequencing technology. The availability of preliminary genomic details of *C. unicolor* SP02 wound facilitates genome-scale understanding of its biology and provides valuable genomic and genetic resources for the investigation of lignocellulosic degradation of the Cerrena genus. The *C. unicolor* SP02 genome contains genes encoding wide carbohydrate-active enzymes of ligninolytic, cellulolytic, hemicellulolytic, and pectinolytic abilities. SP02 encodes almost the full enzymatic portfolio for lignin degradation, notably peroxidases and numerous auxiliary enzymes for the generation of hydrogen peroxide, and is associated with degradation of phenylalanine, styrene, aromatic compounds, aminobenzoate, and benzoate. The cultural characteristics suggest the lignocellulolytic capacity of SP02 is dependent on various factors and encourages the future exploitation of SP02 in agro-waste degradation. In conclusion, *C. unicolor* SP02 is a representative model of white-rot fungi for studying the enzyme machinery involved in the degradation/transformation of lignocellulosic materials. The understanding of the genetic material coding for the lignocellulolytic enzymes will significantly benefit us in genetic manipulation, site-directed mutagenesis, and industrial biotechnology. Further functional studies are underway to evaluate SP02 with respect to its potential in various applications.

## Figures and Tables

**Table 1:** Genome assembly statistics of *C. unicolor* SP02 genome.

Feature Items	Statistics
Contig number	58
Length of genome assembly	42,794,305 bp
C + G content	47.43%
N50	2,483,375 bp
Longest contig	4,365,256 bp
Shortest contig	33,827 bp
Average (bp)	737,832.84 bp
Number of protein-coding genes (CDS)	12,277
Total CDS length	21,069,806 bp
Average CDS length (bp)	1716.2 bp
rRNA number	49
tRNA number	230
other ncRNA number	26

**Table 2:** Enzyme performance in SSF mixtures.

Enzymes	Day	Corn Stalk	Wheat Straw	Rice Straw	Pine Bark
Laccase	3	++	++	++	++
	6	+++	++	++	++
	9	++++	+++	++	++
	12	++++	+++	++++	++
	15	++++	+++	+++	++
	18	++++	+++	+++	++
MnP	3	++	+	+/−	−
	6	++	++	+/−	−
	9	++	++	+/−	+/−
	12	++	++	++	−
	15	++	++	++	−
	18	++	++	++	+/−
LiP	3	+	+/−	+/−	+/−
	6	+	+	+	+/−
	9	+	+	+	+/−
	12	+/−	+	+/−	+
	15	+	+	+/−	+
	18	+/−	+/−	+	+/−
CMCase	3	++	++	++	+
	6	++	++	++	+
	9	++	++	+	+
	12	++	++	++	+
	15	++	++	++	+
	18	++	++	++	+
FPase	3	+	++	+	+
	6	++	+	+	+
	9	+	+	+	+
	12	+	+	+	+
	15	+	+	+	+
	18	+	+	+	+

Note: Performance of enzymes was as described in above 4 materials. Scoring: enzyme activity >100 U/g ++++; 10–100 U/g +++; 1–10 U/g ++; <1 U/g +; <1 U/g and detectable/undetectable in parallel experiment +/−; −undetectable.

**Table 3:** Maximum enzyme activity of *C. unicolor* SP02 on different substrates.

Enzyme (U/g)	Corn Stalk	Wheat Straw	Rice Straw	Pine Bark
Lac	177.26 ± 34.33 (9th day)	15.88 ± 3.83 (15th day)	113.36 ± 37.05 (12th day)	8.91 ± 1.44 (6th day)
MnP	7.04 ± 1.62 (6th day)	2.69 ± 0.47 (9th day)	6.53 ± 2.06 (15th day)	0.07 ± 0.06 (9th day)
LiP	0.73 ± 0.19 (9th day)	0.33 ± 0.04 (12th day)	0.68 ± 0.08 (6th day)	0.43 ± 0.24 (12th day)
CMCase	7.14 ± 1.85 (3rd day)	7.88 ± 1.75 (3rd day)	6.10 ± 2.70 (15th day)	0.22 ± 0.01 (6th day)
FPA	1.10 ± 0.29 (6th day)	1.76 ± 0.35 (3rd day)	0.38 ± 0.16 (3rd day)	0.52 ± 0.11 (3rd day)

**Table 4:** Final consumption of *C. unicolor* SP02 on different substrates.

Final Consumption (%)	Corn Stalk	Wheat Straw	Rice Straw	Pine Bark
Total weight loss	28.52 ± 2.58	24.76 ± 0.81	20.82 ± 0.98	9.23 ± 0.14
Lignin loss	53.82 ± 3.02	46.72 ± 0.93	27.34 ± 4.72	10.45 ± 1.23
Cellulose loss	43.45 ± 0.80	33.76 ± 3.64	36.89 ± 3.39	10.51 ± 1.57
Hemicellulose loss	53.90 ± 1.11	44.00 ± 3.83	31.88 ± 3.91	16.38 ± 2.90

## Data Availability

The data presented in this study are shown in the manuscript published here. Raw data are available upon request from the corresponding author.

## Supplementary Materials

The following are available online at https://www.mdpi.com/article/10.3390/jof7100835/s1, Table S1: Additional file of CAZyme-coding genes, Table S2: Additional file of flavin-containing Monooxygenases, Table S3: Additional file of cytochrome P450s, Table S4: Additional file of gene_ID, Table S5: Additional file of repeat region analysis.
